# Strategy intervention for the evolution of fairness

**DOI:** 10.1371/journal.pone.0196524

**Published:** 2018-05-02

**Authors:** Yanling Zhang, Feng Fu

**Affiliations:** 1 Key Laboratory of Knowledge Automation for Industrial Processes of Ministry of Education, School of Automation and Electrical Engineering, University of Science and Technology Beijing, Beijing, China; 2 Department of Mathematics, Dartmouth College, Hanover, NH, United States of America; 3 Department of Biomedical Data Science, Geisel School of Medicine, Dartmouth College, Hanover, NH, United States of America; Universidad Loyola Andalucia, SPAIN

## Abstract

The ‘irrational’ preference for fairness has attracted increasing attention. Although previous studies have focused on the effects of spitefulness on the evolution of fairness, they did not consider non-monotonic rejections shown in behavioral experiments. In this paper, we introduce a non-monotonic rejection in an evolutionary model of the Ultimatum Game. We propose strategy intervention to study the evolution of fairness in general structured populations. By sequentially adding five strategies into the competition between a fair strategy and a selfish strategy, we arrive at the following conclusions. First, the evolution of fairness is inhibited by altruism, but it is promoted by spitefulness. Second, the non-monotonic rejection helps fairness overcome selfishness. Particularly for group-structured populations, we analytically investigate how fairness, selfishness, altruism, and spitefulness are affected by population size, mutation, and migration in the competition among seven strategies. Our results may provide important insights into understanding the evolutionary origin of fairness.

## Introduction

In the last thirty years, there has been substantial progress in understanding the evolution of fairness by studying the Ultimatum Game (UG). In a typical UG, a proposer and a responder allocate a fixed sum of money. The proposer makes a proposal about how to allocate the money and the responder decides to accept the proposal or not. If the proposal is accepted, both of them are paid accordingly. Otherwise, neither of them is paid. If each player in the game tries to maximize his own payoff, the responder should accept any non-zero offers [1]. In such case, the proposer should offer the minimum allowable proportion of the sum to the responder. However, this prediction contradicts with nearly all experimental observations, in which responders usually reject offers less than 30% of the sum and the most common offer of proposers is 50% of the sum (see reviews [2, 3]).

Here, we denote an offer less than 50% of the sum as a low offer and an offer more than 50% as a high offer. Most experiments which are confined in typical student populations have shown that players reject low offers and rarely reject high offers. However, some experiments which are confined in non-student populations have found the rejection of high offers [4–8]. The rejection of an offer can be regarded as the behavior of costly punishment [9–11], because the responder voluntarily suffers the cost of the ‘offer’ to cause the proposer not to obtain the remaining amount. Some investigations argue that such rejection is motivated by individuals’ prosocial preference for fairness [12, 13]. The preference for spitefulness has been found to be another potential motivation [14–16]. In this paper, we will pay attention to the effects of spitefulness on the evolution of fairness.

Recently, the effects of spitefulness on the evolution of fairness have been studied by one evolutionary game model [17]. This model has focused on the replicator dynamics of four discrete strategies in infinite populations, which represent selfishness, fairness, altruism, and spitefulness, respectively. It has implicitly shown spitefulness promotes the evolution of fairness in infinite populations under certain conditions. Unlike the previous work, we will introduce non-monotonic rejections which reject low offers and high offers in finite populations, which have been found in behavioral experiments [6, 7]. Besides the four strategies in the previous model, our model will adopt three new strategies, which represent altruism, spitefulness, and fairness, respectively. Moreover, we will use strategy intervention to explicitly study how spitefulness and altruism influence the evolution of fairness in finite populations. Specifically, we start by studying the competition between a selfish strategy and a fair strategy, and then add five strategies to them in sequence. Our study will go from the two-strategy competition to the seven-strategy competition. In particular, the four-strategy competition in our model can recover to the previous model but with finite populations.

The evolution of fairness has been widely studied by evolutionary game models [18–29] and many other models [30–32] (the model in [30] is based on the notion of “cooperative equilibrium” first introduced in [33, 34]). Evolutionary dynamics could characterize genetic evolution and cultural evolution [35–44], both of which have been used to account for the UG experimental phenomena [4, 45]. The deterministic evolutionary dynamics shows that fairness cannot evolve in infinitely large well-mixed populations without additional mechanisms [18]. To promote the evolution of fairness, many additional mechanisms have been proposed: reputation (the proposer knows what offers the responder has accepted in the past) [18], empathy (individuals make offers which they would be prepared to accept) [19], spatial structures [20–26], and repeated interactions [27]. Without these additional mechanisms, fairness has also been found to emerge in finite populations even with the well-mixed structure [28]. In this paper, we will focus on the evolution of fairness in structured populations of finite size.

We will analyze our model based on the well-known Tarnita-*σ* condition [46], which is a simple and general condition for strategy *k* ∈ {1, 2, ⋯, *S*} to be favored by natural selection. Specifically, the average frequency of strategy *k* over the stationary distribution is greater than 1/*S* under weak selection if and only if

$$\begin{array}{c} \Gamma_{1}\left(a_{kk}-{\bar{a}}_{**}\right)+\Gamma_{2}\left({\bar{a}}_{k*}-{\bar{a}}_{*k}\right)+\Gamma_{3}\left({\bar{a}}_{k*}-\bar{a}\right)>0 \end{array}$$ (1)

where *a*_*ij*_ is the payoff of an individual using strategy *i* when interacting with an individual using strategy *j*, ${\bar{a}}_{**}=\frac{1}{S}\sum_{i=1}^{S}a_{ii}$, ${\bar{a}}_{k*}=\frac{1}{S}\sum_{i=1}^{S}a_{ki}$, ${\bar{a}}_{*k}=\frac{1}{S}\sum_{i=1}^{S}a_{ik}$, and $\bar{a}=\frac{1}{S^{2}}\sum_{i=1}^{S}\sum_{j=1}^{S}a_{ij}$. The condition implicates that strategy selection is simply the sum of two competition terms. One is evaluated in states of pairwise strategies and the other one is evaluated in the state of all strategies with the same frequency. The evolutionary process has a great number of possible states, each of which should indicate strategies and locations of all individuals. Therefore, it is surprising for the condition to be so simple. The condition holds for a large class of population structures and update rules satisfying some mild assumptions. The population structure could involve interactions between neighbor nodes on a graph [47], or between individuals of the same group, phenotype, or set [48–50]. The update rule could be the Moran process, the Wright-Fisher process, or the pairwise comparison process.

We will investigate the impacts of altruism and spitefulness on the evolution of fairness in general structured populations. In such case, the unknown parameters Γ_1_, Γ_2_, and Γ_3_ in the Tarnita-*σ* condition do not have to be calculated. Particularly for group-structured populations, we will quantitatively analyze how the evolution of selfishness, fairness, altruism, and spitefulness is influenced by population size, mutation, and migration, respectively. Moreover, we will compare the results between the Moran process and the Wright-Fisher process. A necessary premise for these analyses is the calculation of Γ_1_, Γ_2_, and Γ_3_. We will calculate them based on the results in the previous literature [51], which have been used to analyze the multiple-strategy competition in general models. From a long-term perspective, the group-structured population without migration evolves just like the one-group population, because the absorbing state is that all individuals are located in the same group. The one-group population can also be seen as the well-mixed population. Accordingly, our results in the absence of migration are appropriate for well-mixed populations.

## Model and method

**Fig 1:**
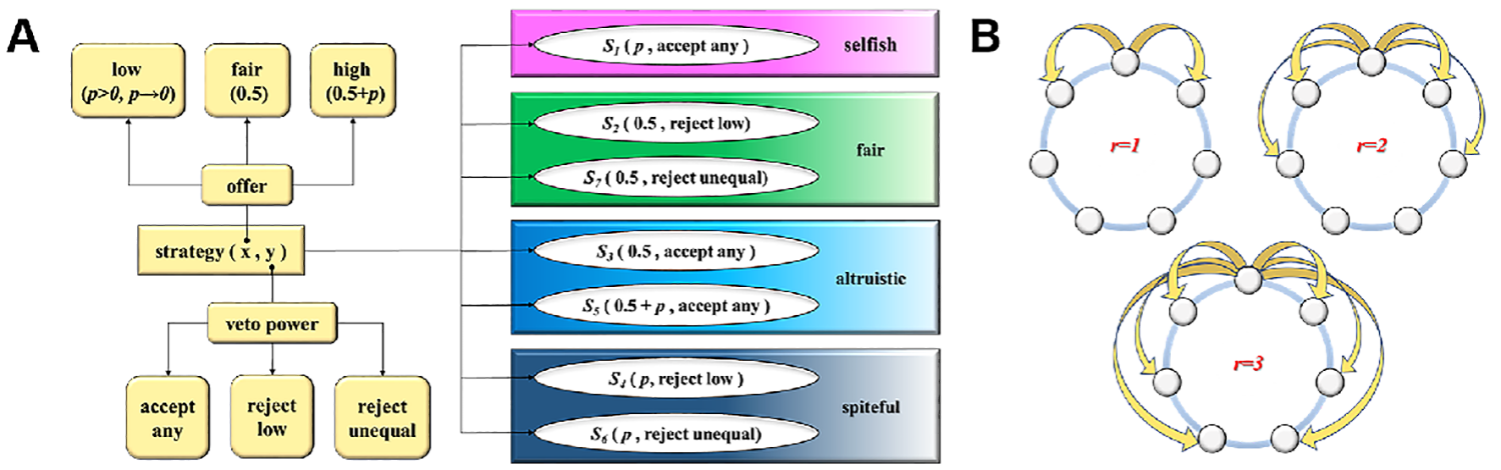
Model schematic. (A) The simplified version of the UG with seven representative discrete strategies. (B) Migration of the range *r* when seven groups are located in a circle.

In the UG, the proposer has to divide a certain amount of money, say 1, with the responder who can accept or reject the split. If the responder accepts the split, the money is shared accordingly; if not, both individuals remain empty handed. We focus on a simplified version of the UG in Fig 1. Proposers have three kinds of offers: the fair offer (0.5), a low offer (*p* > 0, *p* → 0), and a high offer (0.5 + *p*). The first one is an equal offer for the proposer and the responder, whereas the latter two are unequal offers. Some experiments have found that many responders use non-monotonic rejections which reject low offers and high offers [6, 7]. Besides the non-monotonic rejection, we assume that responders have three kinds of veto power: accept any offers (accept any), reject the low offer (reject low), and reject the two unequal offers (reject unequal). A strategy should denote what choice to make as a proposer and what choice to make as a responder. Seven representative discrete strategies will be used: *S*_1_ = (*p*, accept any), *S*_2_ = (0.5, reject low), *S*_3_ = (0.5, accept any), *S*_4_ = (*p*, reject low), *S*_5_ = (0.5 + *p*, accept any), *S*_6_ = (*p*, reject unequal), and *S*_7_ = (0.5, reject unequal).

The strategies *S*_1_, *S*_3_, and *S*_5_ have a common point, i.e., accepting the low offer as the responder. Such behavior has been found to be selfishness or altruism by a behavioral experiment [52]. Selfishness is a desire to maximize one’s own payoff. The strategy *S*_1_ displays selfishness by offering very little as the proposer and accepting any offers as the responder. Altruism is a desire to be kind to opponents using any strategies. The strategies *S*_3_ and *S*_5_ display altruism by giving the opponent a non-low offer and accepting any offers of the opponent. The strategies *S*_2_, *S*_7_, *S*_4_, and *S*_6_ have the common behavior as responders, i.e., rejecting the low offer. This behavior has been found to be fairness or spitefulness by a behavioral experiment [15]. Fairness is a desire to sacrifice one’s own payoff to pursue fairness. For *S*_2_ and *S*_7_, the proposer gives up his priority to play fair to the opponent and the responder punishes the unequal offer of the opponent at a cost. Therefore, the strategies *S*_2_ and *S*_7_ represent fairness. Spitefulness is a desire to obtain an advantageous standing over one’s opponent. For *S*_4_, the proposer is always trying to get a higher payoff than the opponent and the responder will not leave his own payoff below his opponent’s. Therefore, the strategy *S*_4_ represents spitefulness. *S*_6_ is a complex strategy because it does not seem to fully fit in one of the above four preference types. It exhibits spitefulness from the perspective of the proposer, and it exhibits fairness from the perspective of the responder. Therefore, the strategy *S*_6_ represents spitefulness and fairness. If a person causes others to suffer obvious loss by showing spitefulness and cannot help others obtain obvious benefit by showing fairness, we usually remember his spitefulness and neglect his fairness. In this sense, we label *S*_6_ as spitefulness in this paper.

We will first consider general structural populations satisfying the Tarnita-*σ* condition. A given interaction is comprised of two games, in which two individuals play the roles of proposer and responder alternately. The payoff matrix for the simplified version of the UG is shown in Table 1. All interactions accumulate the payoff of individual *i*, *p*_*i*_, and further his fitness, *f*_*i*_ = 1 + *δp*_*i*_, where *δ* is the selection intensity. Mutation may occur during reproduction. With probability *u*, mutation occurs on one of the offspring, and then he equi-probably chooses one of the possible strategies. Otherwise, the offspring inherits the strategy of his parent.

**Table 1:** The payoff matrix for the simplified version of the UG.

	*S*_1_	*S*_2_	*S*_3_	*S*_4_	*S*_5_	*S*_6_	*S*_7_
***S*_1_**(***p***, **accept any**)	1	1/2	3/2-*p*	*p*	3/2	*p*	1/2
***S*_2_**(**0.5**, **reject low**)	1/2	1	1	1/2	1 + *p*	1/2	1
***S*_3_**(**0.5**, **accept any**)	1/2 + *p*	1	1	1/2 + *p*	1 + *p*	1/2 + *p*	1
***S*_4_**(***p***, **reject low**)	1-*p*	1/2	3/2-*p*	0	3/2	0	1/2
***S*_5_**(**0.5 + *p***, **accept any**)	1/2	1-*p*	1-*p*	1/2	1	*p*	1/2
***S*_6_**(***p***, **reject unequal**)	1-*p*	1/2	3/2-*p*	0	1-*p*	0	1/2
***S*_7_**(**0.5**, **reject unequal**)	1/2	1	1	1/2	1/2	1/2	1

We will then consider group-structured populations. Specifically, all individuals are distributed over *M* groups which are located in a circle, and an individual only interacts with the others of the same group. The Moran process and the Wright-Fisher process will be studied, respectively. In the Moran process, all individuals compete to reproduce one offspring proportional to their fitness, and then one individual is equi-probably chosen from the whole population to die. In the Wright-Fisher process, all individuals compete to reproduce *N* (population size) offspring proportional to their fitness, and then they all are replaced by the newborn offspring. Besides mutation, migration is also introduced in our model. With probability 1 − *v*, the offspring remains in his parent’s group. Otherwise, he moves to a new group according to the migration pattern of the range *r* shown in Fig 1. For the migration range *r*, all possible displacements generated by a single-step migration are contained in the set Ω(*r*) = {1, 2, ⋯, *r*}. We assume that all elements of Ω(*r*) are performed equi-probably.

The comparison among selfishness, fairness, altruism, and spitefulness is based on *f*_1_, *f*_2_, *f*_3_, and *f*_4_ in Table 2. Take selfishness and fairness for example. Selfishness has an advantage over fairness if *f*_1_ > *f*_2_, the reverse holds if *f*_1_ < *f*_2_, and they compete equally if *f*_1_ = *f*_2_. Let $F_{k}=\Gamma_{1}\left(a_{kk}-{\bar{a}}_{**}\right)+\Gamma_{2}\left({\bar{a}}_{k*}-{\bar{a}}_{*k}\right)+\Gamma_{3}\left({\bar{a}}_{k*}-\bar{a}\right)$. When selfishness, fairness, altruism, or spitefulness is exhibited by a single strategy, we assume *f*_*i*_ = *F*_*i*_ with *i* ∈ {1, 2, 3, 4}. Under weak selection, all possible strategies have similar frequencies. To guarantee that the comparison proceeds on the same scale, we assume *f*_2_ = (*F*_2_ + *F*_7_)/2, *f*_3_ = (*F*_3_ + *F*_5_)/2, or *f*_4_ = (*F*_4_ + *F*_6_)/2 when fairness, altruism, or spitefulness is exhibited by two strategies.

**Table 2:** *f*_1_, *f*_2_, *f*_3_, and *f*_4_.

	*f*_1_ = *F*_1_	*f*_2_ = *F*_2_ or *f*_2_ = (*F*_2_ + *F*_7_)/2	*f*_3_ = *F*_3_ or *f*_3_ = (*F*_3_ + *F*_5_)/2	*f*_4_ = *F*_4_ or *f*_4_ = (*F*_4_ + *F*_6_)/2
**three-strategy competition**	$\frac{3\left(1-2p\right)\Gamma_{2}+\left(1-3p\right)\Gamma_{3}}{9}$	$-\frac{\Gamma_{3}}{18}$	$-\frac{6\left(1-2p\right)\Gamma_{2}+\left(1-6p\right)\Gamma_{3}}{18}$	
**four-strategy competition**	$\frac{\Gamma_{1}}{4}$	$\frac{\Gamma_{1}}{4}$	$\frac{\Gamma_{1}-2\left(1-2p\right)\Gamma_{2}+2p\Gamma_{3}}{4}$	$\frac{-3\Gamma_{1}+2\left(1-2p\right)\Gamma_{2}-2p\Gamma_{3}}{4}$
**five-strategy competition**	$\frac{10\Gamma_{1}+10\Gamma_{2}+3\Gamma_{3}}{50}$	$\frac{5\Gamma_{1}+10p\Gamma_{2}-\left(1-5p\right)\Gamma_{3}}{25}$	$\frac{10\Gamma_{1}-10\left(2-p\right)\Gamma_{2}-\left(2-50p\right)\Gamma_{3}}{50}$	$\frac{-40\Gamma_{1}+10\left(3-4p\right)\Gamma_{2}+\left(3-20p\right)\Gamma_{3}}{50}$
**six-strategy competition**	$\frac{12\Gamma_{1}+12p\Gamma_{2}+\left(1+6p\right)\Gamma_{3}}{36}$	$\frac{12\Gamma_{1}+12p\Gamma_{2}+\left(1+6p\right)\Gamma_{3}}{36}$	$\frac{24\Gamma_{1}-36\left(1-p\right)\Gamma_{2}-\left(1-18p\right)\Gamma_{3}}{72}$	$\frac{-48\Gamma_{1}+\left(36-60p\right)\Gamma_{2}-\left(1+30p\right)\Gamma_{3}}{72}$
**seven-strategy competition**	$\frac{2\Gamma_{1}+2p\Gamma_{2}+p\Gamma_{3}}{7}$	$\frac{8\Gamma_{1}+4p\Gamma_{2}+\left(1+2p\right)\Gamma_{3}}{28}$	$\frac{4\Gamma_{1}-6\left(1-p\right)\Gamma_{2}+3p\Gamma_{3}}{14}$	$\frac{-20\Gamma_{1}+4\left(3-5p\right)\Gamma_{2}-\left(1+10p\right)\Gamma_{3}}{28}$

Three-strategy competition is the competition of *s*_1_, *s*_2_, *s*_3_, four-strategy competition is the competition of *s*_1_, *s*_2_, *s*_3_, *s*_4_, five-strategy competition is the competition of *s*_1_, *s*_2_, *s*_3_, *s*_4_, *s*_5_, six-strategy competition is the competition of *s*_1_, *s*_2_, *s*_3_, *s*_4_, *s*_5_, *s*_6_, and seven-strategy competition is the competition of *s*_1_, *s*_2_, *s*_3_, *s*_4_, *s*_5_, *s*_6_, *s*_7_.

## Results

### Structured populations satisfying the Tarnita-*σ* condition

**Fig 2:**
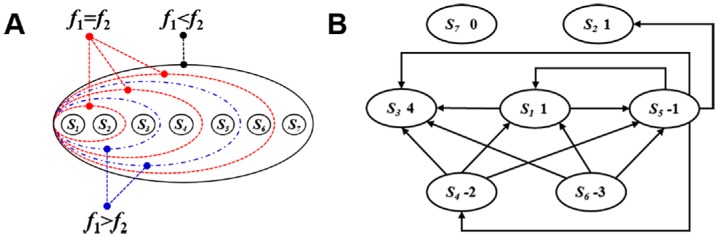
Evolutionary dynamics in structured populations satisfying the Tarnita-*σ* condition. A: The competition of selfishness (*S*_1_) and fairness (*S*_2_ and *S*_7_) is influenced by altruism (*S*_3_ and *S*_5_) and spite (*S*_4_ and *S*_6_). B: For the seven-strategy competition, the scores of *S*_1_, *S*_2_, *S*_3_, *S*_4_, *S*_5_, *S*_6_, and *S*_7_ are calculated.

When the selfish strategy *S*_1_ and the fair strategy *S*_2_ coexist in the population, they compete equally with each other, i.e., *f*_1_ = *f*_2_. This is because their payoffs are identical for all population states. By sequentially adding *S*_3_, *S*_4_, *S*_5_, *S*_6_, and *S*_7_, we show how the competition between selfishness and fairness is influenced by altruism and spitefulness in Fig 2. When the altruistic strategy *S*_3_ with the fair offer is introduced into the population, the selfish strategy *S*_1_ gains an advantage over the fair strategy *S*_2_, i.e., *f*_1_ > *f*_2_. This advantage can be removed, i.e., *f*_1_ = *f*_2_, by introducing the spiteful strategy *S*_4_ which rejects the low offer. When we continue to introduce the altruistic strategy *S*_5_ with the high offer, the advantage of *S*_1_ over *S*_2_ shows up again. Then the advantage is removed when we continue to introduce the spiteful strategy *S*_6_ which rejects unequal offers. Accordingly, the evolution of fairness is inhibited by altruism, but it is promoted by spitefulness. The reason is twofold: Compared with *S*_1_, *S*_2_ obtains less payoffs from two altruistic strategies and more payoffs from two spiteful strategies. It can be understood intuitively by comparing the row sum of *S*_1_ with that of *S*_2_ in Table 1. When the fair strategy *S*_7_ which rejects unequal offers is introduced, the total row sum of *S*_2_ and *S*_7_ is greater than twice of *S*_1_’s row sum in Table 1. Therefore, fairness which is measured by the average of *S*_2_ and *S*_7_ first gains an advantage over selfishness, i.e., *f*_2_ > *f*_1_, in the seven-strategy competition. This means that the non-monotonic rejection helps fairness overcome selfishness.

As shown in Table 2, the increase of *p* induces the frequencies of selfishness, fairness, and altruism to increase in the seven-strategy competition. However, the increase of *p* induces the frequency of spitefulness to decrease. Assume *X* is one of *S*_1_, *S*_4_, *S*_5_, and *S*_6_, then a larger *p* causes the proposer using *X* to obtain a less payoff once the responder using another strategy *Y* accepts the offer. Here, we define that *X* gives *Y* one score and draw one arrow which goes from *X* to *Y* in Fig 2. Fig 2 shows that *S*_1_ gives two strategies two scores and three strategies give *S*_1_ three scores. The score of the selfish strategy *S*_1_ is positive, meaning that the increase of *p* helps *S*_1_ obtain more payoffs. Accordingly, the increase of *p* raises the frequency of selfishness. By using a similar analysis, we can arrive at the following conclusions. The total score of two fair strategies *S*_2_ and *S*_7_ is positive, and thus the increase of *p* raises the frequency of fairness. The total score of two altruistic strategies *S*_3_ and *S*_5_ is positive, and thus the increase of *p* raises the frequency of altruism. The total score of two spiteful strategies *S*_4_ and *S*_6_ is negative, and thus the increase of *p* reduces the frequency of spitefulness. For the rest of the paper, we will only focus on the case of *p* = 0.01.

### Group-structured populations

The seven-strategy competition is also investigated in group-structured populations. For the Moran process and the Wright-Fisher process, the average frequency of strategy *k* ∈ {1, 2, ⋯, *S*} over the stationary distribution under weak selection (*δ* → 0), 〈*x*_*k*_〉_*δ* → 0_, is given by

$$\begin{array}{c} {\left\langle x_{k}\right\rangle }_{\delta \to 0}=\frac{1}{S}+\delta \frac{1-u}{Nu}\left(\Gamma_{1}\left(a_{kk}-{\bar{a}}_{**}\right)+\Gamma_{2}\left({\bar{a}}_{k*}-{\bar{a}}_{*k}\right)+\Gamma_{3}\left({\bar{a}}_{k*}-\bar{a}\right)\right) \end{array}$$ (2)

in which Γ_1_, Γ_2_, and Γ_3_ are unknown. Let *I*_*ij*_ be the total number of interactions between strategy *i* and strategy *j*. Then Γ_1_, Γ_2_, and Γ_3_ can be expressed by 〈*x*_*i*_ *I*_*jk*_〉_0_ which is the probability-weighted average of *x*_*i*_ *I*_*jk*_ over all possible steady states under neutral selection:

$$\begin{array}{c} \Gamma_{1}=S\left({\left\langle x_{1}I_{22}\right\rangle }_{0}-{\left\langle x_{1}I_{23}\right\rangle }_{0}\right)\;\;\;\Gamma_{2}=S\left({\left\langle x_{1}I_{12}\right\rangle }_{0}-{\left\langle x_{1}I_{23}\right\rangle }_{0}\right)\;\;\;\Gamma_{3}=S^{2}{\left\langle x_{1}I_{23}\right\rangle }_{0} \end{array}$$ (3)

**Fig 3:**
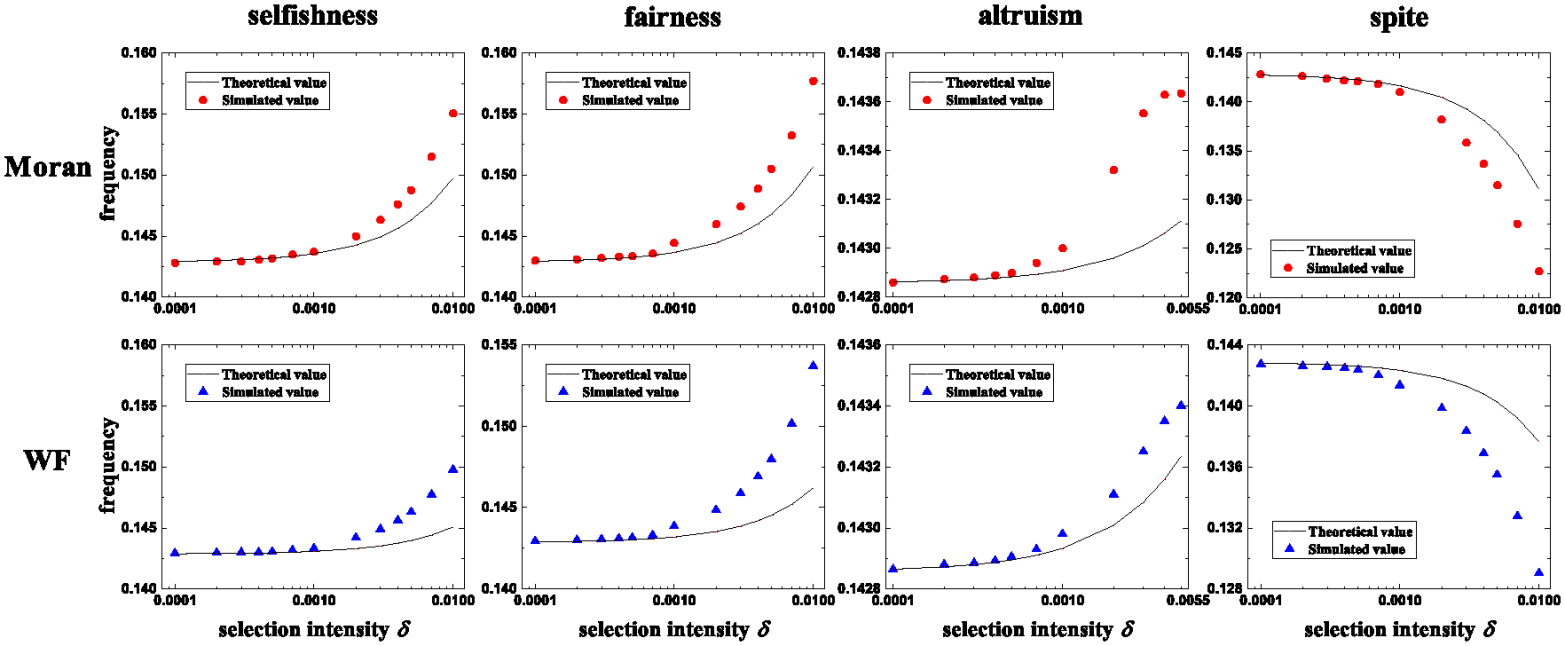
Comparison of theoretical results and simulated results in the Moran process (first row) and the Wright-Fisher process (second row). For the frequencies of selfishness (first column), fairness (second column), altruism (third column), and spite (fourth column), the difference of the theoretical values and the simulated values (averaged over 5 × 10^8^ − 10^6^ generations) is negligible when the selection intensity *δ* is sufficiently small, and is no longer negligible for other *δ*. Parameters: *N* = 50, *M* = 7, *u* = 0.1, *v* = 0.1, *r* = 1, and *p* = 0.01.

More details can be obtained from pages 1 − 2 of SI (Supplementary Information) in Ref. [51]. The calculation of 〈*x*_*i*_ *I*_*jk*_〉_0_ can be transformed into calculating the probabilities that three randomly chosen individuals use given strategies and are located in given groups (please refer to pages 3 − 4 of SI in Ref. [51] for more details). These probabilities have been calculated for the Moran process and the Wright-Fisher process (please refer to pages 5 − 10 and pages 10 − 16 of SI in Ref. [51] for more details). We first take the known values of these probabilities into the expression of 〈*x*_*i*_ *I*_*jk*_〉_0_. Then we obtain the precise values of Γ_1_, Γ_2_, and Γ_3_ according to Eq (3), which are summarized in Table 3. The expression of *f*(*x*) in Table 3, which corresponds to the migration range *r*, is given by

$$\begin{array}{ccc} f(x) & = & \frac{1}{M-1}\sum_{j=1}^{M-1}\cos\frac{2\pi jx}{M}\;\text{if}\;r=\frac{M}{2}\;\text{for}\;\text{even}\;M\\ f(x) & = & \frac{1}{r}\sum_{j=1}^{r}\cos\frac{2\pi jx}{M}\;\text{else}\;\text{if}\;r=1,2,\cdots ,\left\lfloor \frac{M}{2}\right\rfloor \end{array}$$ (4)

where $\left\lfloor \frac{M}{2}\right\rfloor$ is the greatest integer no greater than $\frac{M}{2}$. These results hold for arbitrary population sizes, non-zero mutation probabilities, migration probabilities, migration ranges, and group numbers. Fig 3 shows that analytical results agree well with simulated results for sufficiently small *δ* and display obvious disagreement with simulated results for other *δ*.

**Table 3:** Γ_1_, Γ_2_ and Γ_3_ for the Moran process ($\Gamma_{1}^{Mo}$, $\Gamma_{2}^{Mo}$, and $\Gamma_{3}^{Mo}$ respectively), and Γ_1_, Γ_2_ and Γ_3_ for the Wright-Fisher process ($\Gamma_{1}^{WF}$, $\Gamma_{2}^{WF}$, and $\Gamma_{3}^{WF}$ respectively), where Φ_*i*_(*f*(*x*)), Ψ_*i*_(*f*(*x*)), $\Phi_{i}^{{}^{\prime }}\left(f\left(x\right)\right)$, and $\Psi_{i}^{{}^{\prime }}\left(f\left(x\right)\right)$ are abbreviated as Φ_*i*_, $\Psi_{i},\Phi_{i}^{{}^{\prime }}$, and $\Psi_{i}^{{}^{\prime }}$.

$\Gamma_{1}^{Mo}$	$\left(N-1\right)\left(N-2\right)/\left(3M\right)\sum_{x=1}^{M}\left(-2\Phi_{1}\Psi_{2}-\Phi_{4}\alpha_{1}+3\Psi_{2}\right)$
$\Gamma_{2}^{Mo}$	$\left(N-1\right)/\left(3M\right)\sum_{x=1}^{M}\left(3\Psi_{1}-3\Psi_{2}+\left(N-2\right)\left(-2\Phi_{1}\Psi_{2}-\Phi_{4}\alpha_{1}+\Phi_{2}\Psi_{2}+\Phi_{3}\Psi_{1}+\Phi_{5}\alpha_{1}\right)\right)$
$\Gamma_{3}^{Mo}$	$\left(N-1\right)\left(N-2\right)/\left(3M\right)\sum_{x=1}^{M}\left(3\Psi_{1}-3\Psi_{2}+2\left(2\Phi_{1}\Psi_{2}+\Phi_{4}\alpha_{1}-\Phi_{2}\Psi_{2}-\Phi_{3}\Psi_{1}-\Phi_{5}\alpha_{1}\right)\right)$
$\Gamma_{1}^{WF}$	$\left(N-1\right)\left(N-2\right)/\left(3M\right)\sum_{x=1}^{M}\left(-\Phi_{2}^{{}^{\prime }}\left(2N\Psi_{2}^{{}^{\prime }}+N\alpha_{1}^{{}^{\prime }}-2\right)+\Phi_{3}^{{}^{\prime }}\left(2N\Psi_{2}^{{}^{\prime }}+N-2\right)\right)$
$\Gamma_{2}^{WF}$	$\left(N-1\right)/\left(3M\right)\sum_{x=1}^{M}\left(\Phi_{1}^{{}^{\prime }}\left(2N\Psi_{1}^{{}^{\prime }}+N-2\right)-\left(N-2\right)\Phi_{2}^{{}^{\prime }}\left(2N\Psi_{2}^{{}^{\prime }}+N\alpha_{1}^{{}^{\prime }}-2\right)-\Phi_{3}^{{}^{\prime }}\left(2N\Psi_{2}^{{}^{\prime }}+N-2\right)+\left(N-2\right)\Phi_{3}^{{}^{\prime }}\left(N\Psi_{2}^{{}^{\prime }}+N\Psi_{1}^{{}^{\prime }}+N\alpha_{1}^{{}^{\prime }}-2\right)\right)$
$\Gamma_{3}^{WF}$	$\left(N-1\right)\left(N-2\right)/\left(3M\right)\sum_{x=1}^{M}\left(\Phi_{1}^{{}^{\prime }}\left(2N\Psi_{1}^{{}^{\prime }}+N-2\right)+2\Phi_{2}^{{}^{\prime }}\left(2N\Psi_{2}^{{}^{\prime }}+N\alpha_{1}^{{}^{\prime }}-2\right)-\Phi_{3}^{{}^{\prime }}\left(2N\Psi_{2}^{{}^{\prime }}+N-2\right)-2\Phi_{3}^{{}^{\prime }}\left(N\Psi_{2}^{{}^{\prime }}+N\Psi_{1}^{{}^{\prime }}+N\alpha_{1}^{{}^{\prime }}-2\right)\right)$

$\alpha_{1}=\frac{1-u}{1+(N-1)u}$, $\Phi_{1}\left(f\right)=\frac{\left(1-u)(2-v(1-f)\right)}{2+\left(N-2\right)u+\frac{2(N-2)(1-u)v}{3}\left(1-f\right)}$, $\Phi_{2}\left(f\right)=\frac{2-u-v(1-f)}{2+\frac{2(N-2)u}{3}+\frac{(N-2)(2-u)v}{3}\left(1-f\right)}$, $\Phi_{3}\left(f\right)=\frac{\left(1-u)(2-v(1-f)\right)}{2+\frac{2(N-2)u}{3}+\frac{(N-2)(2-u)v}{3}\left(1-f\right)}$, $\Phi_{4}\left(f\right)=\frac{\left(1-u)(1-v(1-f)\right)}{1+\frac{(N-2)u}{2}+\frac{(N-2)(1-u)v}{3}\left(1-f\right)}$, $\Phi_{5}\left(f\right)=\frac{\left(2-u)(1-v(1-f)\right)}{2+\frac{2(N-2)u}{3}+\frac{(N-2)(2-u)v}{3}\left(1-f\right)}$, $\Psi_{1}\left(f\right)=\frac{1-v(1-f)}{1+(N-1)v(1-f)}$, $\Psi_{2}\left(f\right)=\frac{\left(1-u)(1-v(1-f)\right)}{1+(N-1)u+(N-1)(1-u)v(1-f)}$, $\Psi_{1}^{{}^{\prime }}\left(f\right)=\frac{1}{N-\left(N-1\right){\left(1-v\left(1-f\right)\right)}^{2}}$, $\Psi_{2}^{{}^{\prime }}\left(f\right)=\frac{1}{N-\left(N-1\right){\left(1-u\right)}^{2}{\left(1-v\left(1-f\right)\right)}^{2}}$, $\alpha_{1}^{{}^{\prime }}=\frac{1}{N-\left(N-1\right){\left(1-u\right)}^{2}}$, $\Phi_{1}^{{}^{\prime }}\left(f\right)=\frac{{\left(1-v\left(1-f\right)\right)}^{2}}{N^{2}-\left(N-1\right)\left(N-2\right){\left(1-v\left(1-f\right)\right)}^{2}}$, $\Phi_{2}^{{}^{\prime }}\left(f\right)=\frac{{\left(1-u\right)}^{3}{\left(1-v\left(1-f\right)\right)}^{2}}{N^{2}-\left(N-1\right)\left(N-2\right){\left(1-u\right)}^{3}{\left(1-v\left(1-f\right)\right)}^{2}}$, $\Phi_{3}^{{}^{\prime }}\left(f\right)=\frac{{\left(1-u\right)}^{2}{\left(1-v\left(1-f\right)\right)}^{2}}{N^{2}-\left(N-1\right)\left(N-2\right){\left(1-u\right)}^{2}{\left(1-v\left(1-f\right)\right)}^{2}}$.

**Fig 4:**
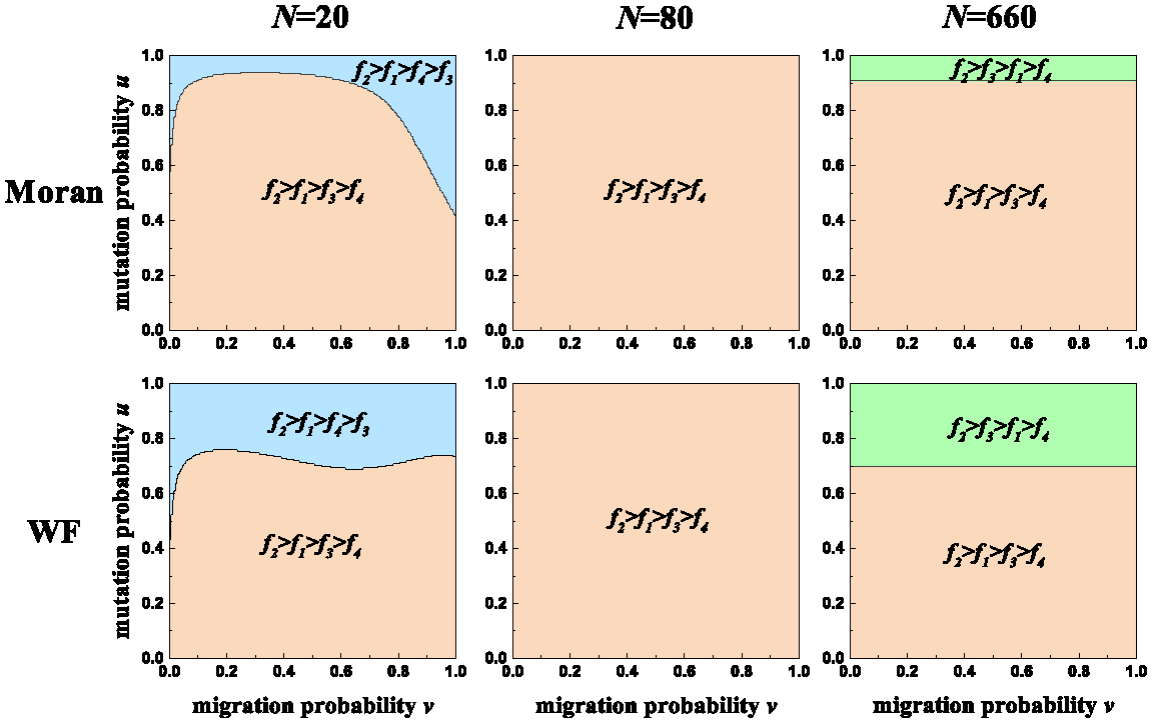
For different *N*, comparison of *f*_1_, *f*_2_, *f*_3_, and *f*_4_ over the area spanned by *v* and *u*. For the Moran process (first row) and the Wright-Fisher process (second row), there are two phenomena *f*_2_ > *f*_1_ > *f*_3_ > *f*_4_ and *f*_2_ > *f*_1_ > *f*_4_ > *f*_3_ in the small population of *N* = 20; there only remains one phenomenon *f*_2_ > *f*_1_ > *f*_3_ > *f*_4_ in the moderate population of *N* = 80; *f*_2_ > *f*_1_ > *f*_3_ > *f*_4_ remains and there appears a new phenomenon *f*_2_ > *f*_3_ > *f*_1_ > *f*_4_ in the large population of *N* = 660. Parameters: *M* = 7, *r* = 1, and *p* = 0.01.

We show the effects of the population size on the seven-strategy competition in Fig 4. Irrespective of the population size, fairness has a higher frequency than the other three behaviors. Accordingly, the population size cannot impact the dominance of fairness. In small populations, selfishness has the second highest frequency. Meanwhile, spitefulness has an advantage over altruism or the opposite holds, i.e., *f*_2_ > *f*_1_ > *f*_4_ > *f*_3_ or *f*_2_ > *f*_1_ > *f*_3_ > *f*_4_. In moderate populations, the former disappears and the latter holds for the whole area spanned by *v* and *u*. In large populations, the (*v*, *u*) area for the latter diminishes. Meanwhile, a new phenomenon appears in which altruism gains an advantage over selfishness, i.e., *f*_2_ > *f*_3_ > *f*_1_ > *f*_4_. Accordingly, we arrive at the following two conclusions. First, the increase of the population size raises the frequency ranking of altruism. It means that a larger population size enhances the evolution of altruism. Second, the increase of the population size reduces the frequency rankings of selfishness and spitefulness. It means that a larger population size weakens the evolution of selfishness and spitefulness. The above results hold for the Moran process and the Wright-Fisher process.

**Fig 5:**
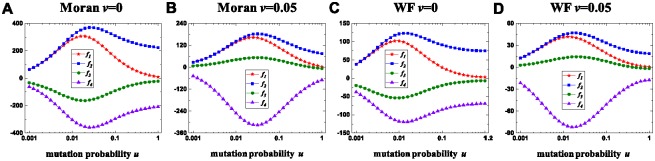
The changing trends of *f*_*i*_, *i* ∈ {1, 2, 3, 4} with *u*. A, C: For the Moran process with *v* = 0 and the Wright-Fisher process with *v* = 0, *f*_1_ and *f*_2_ exhibit inverted U-shaped curves with *u*, but *f*_3_ and *f*_4_ exhibit U-shaped curves with *u*. B, D: For the Moran process with *v* = 0.05 and the Wright-Fisher process with *v* = 0.05, *f*_1_, *f*_2_, and *f*_3_ exhibit inverted U-shaped curves with *u*, but *f*_4_ exhibits a U-shaped curve with *u*. Parameters: *N* = 80, *M* = 7, *r* = 1, and *p* = 0.01.

We demonstrate how mutation influences the seven-strategy competition in Fig 5. Independent of the migration probability *v*, selfishness and fairness exhibit inverted U-shaped curves with the mutation probability *u*. Accordingly, intermediate mutation maximizes selfishness and fairness. Spitefulness exhibits a U-shaped curve with *u* irrespective of *v*. It means that intermediate mutation minimizes spitefulness. Altruism exhibits an inverted U-shaped curve when *v* is intermediate. However, the curve is changed to a U-shaped curve with *u* when *v* is too low or too high (not shown in Fig 5). Therefore, intermediate mutation maximizes altruism for intermediate migration and minimizes altruism otherwise. The above results are appropriate for the Moran process and the Wright-Fisher process.

**Fig 6:**
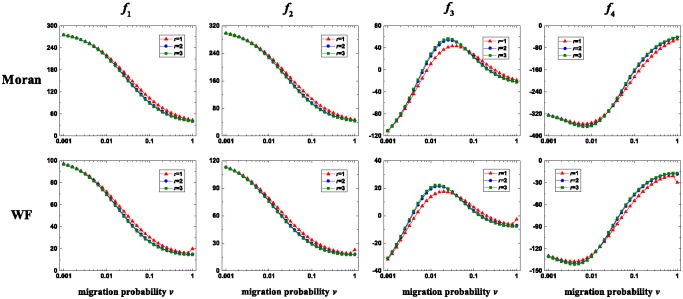
The changing trends of *f*_*i*_, *i* ∈ {1, 2, 3, 4} with *v* for the Moran process (first row) and the Wright-Fisher process (second row) when *u* is low. For all *r*, *f*_1_ (first column) and *f*_2_ (second column) have decreasing trends with *v*. For all *r*, the curve of *f*_3_ (third column) with *v* is inverted U-shaped. For all *r*, The curve of *f*_4_ with *v* (fourth column) has an increasing trend with a small perturbation near *v* = 0.01. When *r* = 1, the curve of *f*_*i*_, *i* ∈ {1, 2, 3, 4} with *v* has a small perturbation near *v* = 1 for the Wright-Fisher process, but not for the Moran process. Parameters: *N* = 80, *M* = 7, *u* = 0.01, and *p* = 0.01.

**Fig 7:**
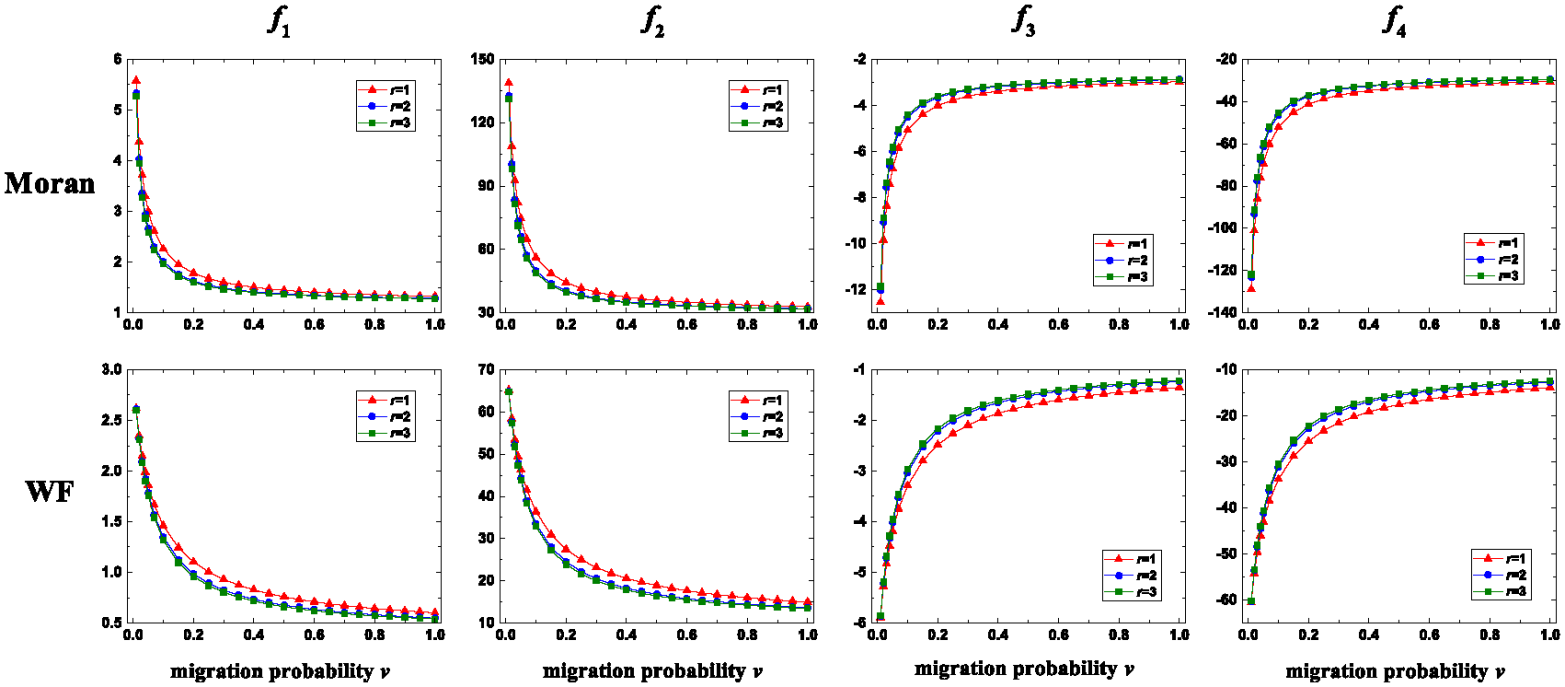
The changing trends of *f*_*i*_, *i* ∈ {1, 2, 3, 4} with *v* for the Moran process (first row) and the Wright-Fisher process (second row) when *u* is high. For all *r*, *f*_1_ (first column) and *f*_2_ (second column) decrease with *v*. For all *r*, *f*_3_ (third column) and *f*_4_ (fourth column) increases with *v*. Parameters: *N* = 80, *M* = 7, *u* = 1, and *p* = 0.01.

We investigate the effects of migration on the seven-strategy competition when *u* is low in Fig 6 and high in Fig 7, respectively. Migration changes selfishness and fairness qualitatively similarly. They both have decreasing trends with *v*, which is independent of *u*. Compared with the case without migration, the existence of migration decreases selfishness and fairness. Therefore, migration inhibits the evolution of selfishness and fairness. The way that altruism changes with *v* is different for low *u* and high *u*. When *u* is low, there exists a moderate *v* which maximizes altruism. When *u* is high, altruism increases with *v*. Compared with the case without migration, the existence of migration increases altruism, and thus migration promotes the evolution of altruism. The way that spitefulness changes with *v* is also different for low *u* and high *u*. When *u* is low, the curve of spitefulness with *v* has an increasing trend with a small perturbation near *v* = 0.01. When *u* is high, spitefulness increases with *v*. Compared with the case without migration, sufficient migration increases spitefulness, and thus it promotes the evolution of spitefulness. The above results are independent of the migration range, and they hold for the Wright-Fisher process and the Moran process. These two update rules have a qualitative difference for the smallest migration range and small *u*. Specifically, the curves of selfishness, fairness, altruism, and spitefulness with *v* have small perturbations at *v* = 1 for the Wright-Fisher process but not for the Moran process.

## Discussion

We have focused on the effects of spitefulness and altruism on the evolution of fairness in general structured populations of finite size. We have used strategy intervention to explicitly study the effects. Specifically, we first study the competition between a selfish strategy and a fair strategy, and then add five strategies to them. Our study goes from the two-strategy competition to the seven-strategy competition. In the two-strategy competition, selfishness competes equally with fairness. The addition of altruism leads to the advantage of selfishness over fairness, and this advantage can be removed by the further addition of spitefulness. When the fair strategy with the non-monotonic rejection is added, fairness gains an advantage over selfishness. Accordingly, we arrive at the following conclusions: 1) The evolution of fairness is inhibited by altruism, but it is promoted by spitefulness; 2) The non-monotonic rejection helps fairness overcome selfishness. The four-strategy competition of our model corresponds to the finite-population version of the previous model [17]. In contrast to our work, the previous model has only studied the replicator dynamics of four strategies in infinite populations. It has implicitly demonstrated that spitefulness promotes the evolution of fairness in infinite populations under certain conditions. Moreover, the previous model has not considered non-monotonic rejections which have been shown in behavioral experiments [6, 7].

Most previous studies about the UG, including the above-mentioned literature [17], have neglected the role of population finiteness in the evolution of fairness. Recently, a stochastic evolutionary model has demonstrated that fairness can evolve in finite populations without any other mechanisms [28]. It indicates that the finiteness of the population matters in the evolution of fairness. In this paper, we have focused on finite populations, including general structured populations and group-structured populations. Particularly for group-structured populations, we have studied the effects of the population size on the seven-strategy competition by the Tarnita-*σ* condition [46]. For the Moran process and the Wright-Fisher process, the population size cannot change the dominance of fairness. A larger population size enhances the evolution of altruism, but it weakens the evolution of selfishness and spitefulness. The unknown parameters in the Tarnita-*σ* condition have been obtained based on the results in the previous literature [51], which have been used to analyze the multiple-strategy competition in general models.

The effects of migration on the evolution of fairness have been previously studied by agent-based simulations [26]. In this paper, we have given the analytic results about how migration and mutation influence the evolution of fairness, selfishness, altruism, and spitefulness. The Moran process and the Wright-Fisher process have the following qualitatively similar results. Intermediate mutation maximizes selfishness and fairness, but it minimizes spitefulness. Intermediate mutation maximizes altruism for intermediate migration and minimizes altruism otherwise. Migration inhibits the evolution of selfishness and fairness, but it promotes the evolution of altruism. Only sufficient migration promotes the evolution of spitefulness. For the smallest migration range and small mutation probabilities, the Moran process and the Wright-Fisher process have the following qualitatively different results. The curves of selfishness, fairness, altruism, and spitefulness with the migration probability *v* have small perturbations at *v* = 1 for the Wright-Fisher process but not for the Moran process.
